# 27-Hydroxycholesterol induces expression of zonula occludens-1 in monocytic cells via multiple kinases pathways

**DOI:** 10.1038/s41598-022-12416-w

**Published:** 2022-05-17

**Authors:** Hyok-rae Cho, Bo-Young Kim, Koanhoi Kim, Dongjun Lee, Seong-Kug Eo, Yonghae Son

**Affiliations:** 1 Department of Pharmacology, School of Medicine, College of Medicine, Pusan National University, 49 Busandaehak-ro, Yangsan, 50612 Korea; 2 Department of Neurosurgery, College of Medicine, Kosin University, Busan, 49267 Korea; 3 Department of Convergence Medicine, School of Medicine, Pusan National University, Yangsan, 50612 Korea; 4 College of Veterinary Medicine and Bio-Safety Research Institute, Chonbuk National University, Iksan, 54596 Korea

**Keywords:** Cell signalling, Inflammation

## Abstract

Zonula occludens (ZO)-1, a tight-junction protein (TJP), is expressed in dendritic cells (DCs) but not in monocytes, and 27-hydroxycholesterol (27OHChol) drives the differentiation of monocytes into DCs. Because the effects of 27OHChol on ZO-1 are not yet clearly defined, we investigated whether 27OHChol induces expression of the TJP. The treatment of human THP-1 monocytic cells with 27OHChol resulted in the elevated transcript levels of ZO-1 but not of ZO-2 or -3. 27OHChol increased the total amount of ZO-1 protein in the cells as well as its level on the cells surface. Cholesterol, however, did not influence expression of ZO-1. And, the expression of ZO-1 protein was mediated by endoplasmic reticulum (ER)-to-Golgi body transport system. Pharmacological kinase inhibition with LY294002 (a PI3K inhibitor), U0126 (a MEK/ERK inhibitor), or PP2 (a Src family kinase inhibitor) resulted in impaired ZO-1 expression at both transcript and protein levels. Drugs that are reported to suppress DC differentiation also inhibited 27OHChol-mediated expression and the localization of ZO-1, indicating the coincidence of ZO-1 upregulation and DC differentiation. These results suggest that ZO-1 is differentially expressed while monocytes differentiate into DCs in the presence of 27OHChol via pathways in which distinct signaling molecules are involved.

## Introduction

Zonula occludens-1 (ZO-1), a type of tight-junction protein (TJP), serves as a scaffold protein to assemble integral membrane proteins, actin cytoskeletons, and cytosolic proteins in tight junctions (TJs)^1,2^. Levels of ZO-1, which is constitutively expressed in almost tissues and several adherent cell types including endothelial cells and smooth muscle cells^3,4^, are altered depending on cell conditions, cell density, or differentiation. Dendritic cells (DCs), but not monocytes, express TJPs including ZO-1^3^, and ZO-1 expression can be induced during this differentiation into dendritic cells. DCs differentiated from THP-1 monocytic cells using cytokines (IL-4, GM-CSF, and TNF-α) express TJPs including ZO-1, occludin, and JAM-A^4^. These findings suggest that levels of ZO-1 increase in response to exogenous stimuli promoting DC differentiation.

Oxysterols are cholesterol metabolites that are generated via enzymatic or non-enzymatic oxidation. The most abundant oxysterol in human blood is 27-hydroxycholesterol (27OHChol), followed by 7-oxygenated cholesterol molecules such as 7-ketocholesterol (7 K), 7α-hydroxycholesterol (7αOHChol), and 7β-hydroxycholesterol (7βOHChol)^5^. The oxysterols not only can induce cell differentiation into either macrophages or DCs but also are involved in ZO-1 expression. 7αOHChol and 27OHChol induce the differentiation of monocytes into DCs displaying a mature phenotype^6–9^, and 7 K induces macrophage differentiation^10^. All three 7-oxygenated cholesterol derivatives (such as 7αOHChol, 7βOHChol, and 7 K) decrease the ZO-1 levels in vascular smooth muscle cells, whereas 7αOHChol alone induces its expression in monocytic cells^11^, indicating the regulation of ZO-1 in the presence of a specific type of oxysterol. However, despite the fact that it is the most abundant oxysterol in vivo, it is unknown whether 27OHChol affects the levels of ZO-1.

In the present study, we examined the effects of 27OHChol on the expression and localization of ZO-1. We also attempted to determine the signaling pathways involved and investigated the association of ZO-1 expression with DC differentiation.

## Results

### Effects of 27OHChol on ZO-1 expression in monocytic cells

**Figure 1:**
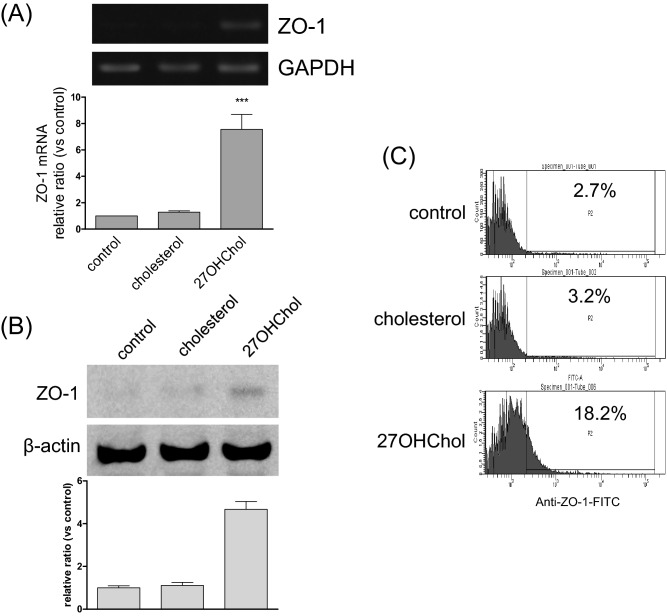
The ZO-1 expression following treatment with 27OHChol. THP-1 cells (1 × 10^6^ cells/60-mm culture dish) were treated for 48 h with 5 µg/ml of cholesterol or 2.5 µg/ml of 27OHChol. (**A**) The transcript levels of ZO-1 were assessed using RT-PCR and real-time PCR. The data are expressed as the mean ± SD (n = 3 replicates/group). ***P < 0.01 versus control. (**B**) ZO-1 was analyzed using Immunoblot. (**C**) After immunostaining ZO-1, the cells were analyzed by flow cytometry. The results are representative of three independent experiments.

To examine whether 27OHChol affected the ZO-1 levels in monocytes, we analyzed the expression of ZO family transcripts using RT-PCR and quantitative real-time PCR in THP-1 cells. Among the ZO-1 family, 27OHChol elevated the transcript levels of ZO-1, but not of ZO-2 or -3, and cholesterol did not influence any ZO-1 family transcript levels (Fig. 1A and Supplementary data 1). In agreement with the results of the RT-PCR and real-time PCR, levels of ZO-1 protein increased with 27OHChol treatment, but not with cholesterol (Fig. 1B). The percentage of cells expressing ZO-1 on the surface increased from 2.7 to 18.7% following treatment with 27OHChol. Cholesterol, however, did not alter the ZO-1 immunoreactivity of the cells (Fig. 1C). We also determined the localization of ZO-1. The immunoreactivity of ZO-1 protein was localized on the cell surface, as visualized via confocal microscopy (Supplementary data 3A). These results suggest that ZO-1 protein is upregulated in the presence of 27OHChol.

**Figure 2:**
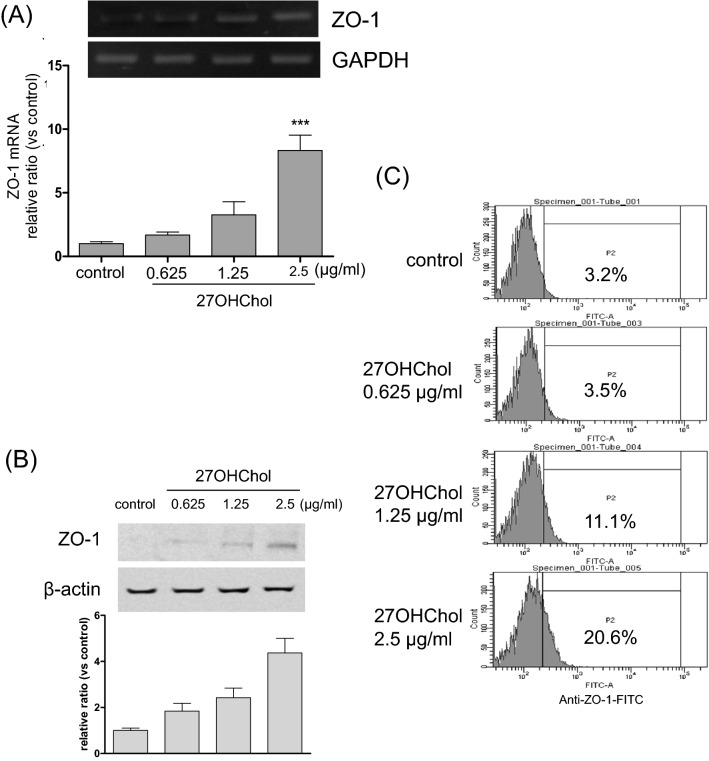
Effects of 27OHChol concentrations on the ZO-1 expression. Monocytic cells were stimulated with the indicated concentrations of 27OHChol for 48 h. (**A**) The transcript levels of the ZO-1 gene were analyzed using quantitative RT-PCR and real-time PCR. The data are expressed as the mean ± SD (n = 3 replicates/group). ***P < 0.01 versus control. (**B**) The proteins harvested from the cells were analyzed by Western blot to detect ZO-1. (**C**) The cells were immunostained with antibodies against ZO-1 and analyzed by flow cytometry. The results are representative of three independent experiments.

**Figure 3:**
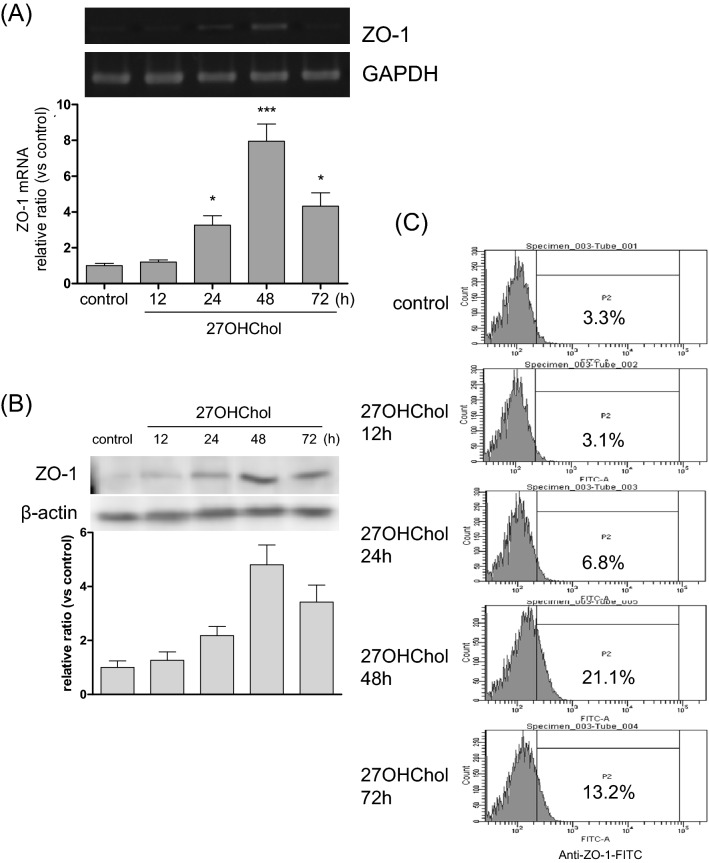
Time-course analyses of the ZO-1 expression following treatment with 27OHChol. THP-1 monocytic cells were stimulated for the indicated durations of time with 2.5 µg/ml of 27OHChol. (**A**) The ZO-1 transcript levels were analyzed using RT-PCR and real-time PCR. The data are expressed as the mean ± SD (n = 3 replicates/group). ***P < 0.01 versus control; *P < 0.1 versus control. (**B**) The ZO-1 protein was detected via Western blot analysis using cell lysates. (**C**) The cells were immunostained with antibodies against ZO-1 and analyzed by flow cytometry. The results are representative of three independent experiments.

We investigated the effects of 27OHChol concentrations on ZO-1 levels. Expression of ZO-1 increased in a dose-dependent manner upon treatment with 27OHChol. 27OHChol, up to a concentration of 2.5 µg/ml, elevated the transcript (Fig. 2A), protein (Fig. 2B), and immunoreactivity levels of ZO-1 (Fig. 2C). We also determined the time-course effects of 27OHChol. The ZO-1 transcripts and protein levels and ZO-1 immunoreactivity on the cell surface reached maximum levels after 48 h of treatment with 27OHChol and decreased thereafter (Fig. 3A–C).

### Involvement of endoplasmic reticulum (ER)-Golgi-transport in ZO-1 expression

**Figure 4:**
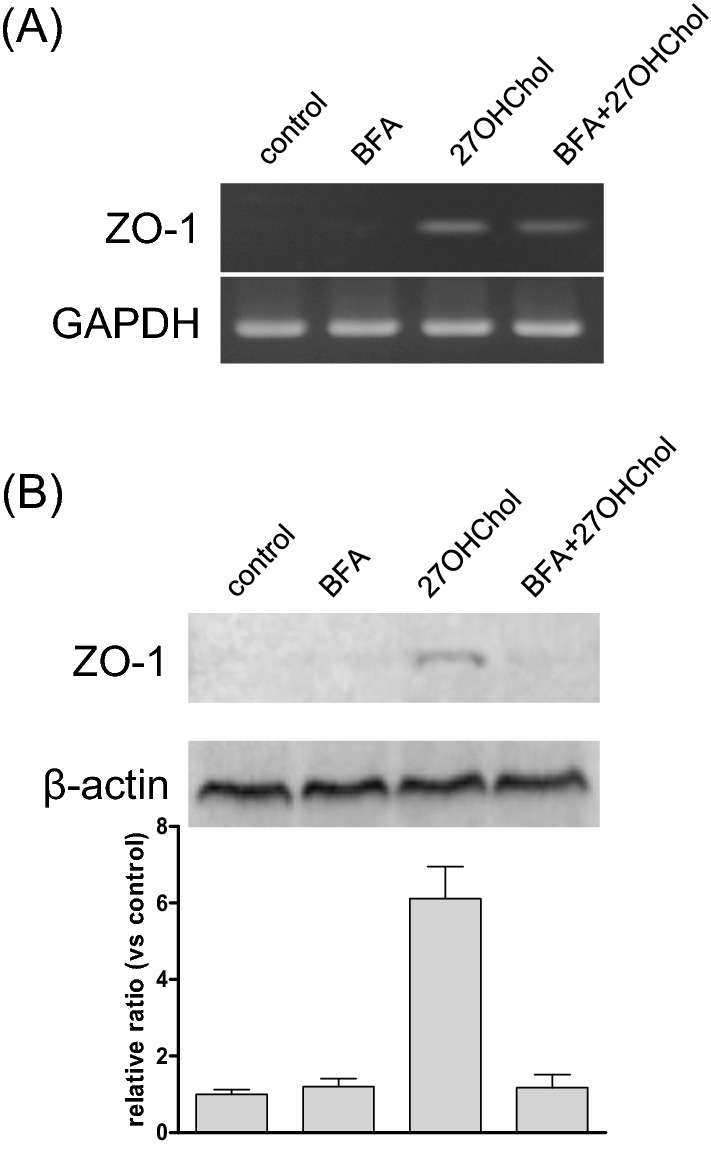
Effects of BFA on the 27OHChol-induced ZO-1 expression. THP-1 cells (1 × 10^6^ cells/60-mm culture dish) were treated for 48 h with 10 µg/ml of BFA and 2.5 µg/ml of 27OHChol. (**A**) The ZO-1 transcript levels were analyzed using RT-PCR. (**B**) The expressed protein of ZO-1 on cell surface was harvested with Cell Surface Protein Isolation Kit, and the isolated protein was analyzed using Western blot. The results are representative of three independent experiments.

We investigated roles of the ER-to-Golgi transport system in the expression of ZO-1 by using BFA. The level of ZO-1 protein on monocytic cells, which was induced by 27OHChol, was downregulated by the treatment with BFA (Fig. 4B). BFA, however, did not affect the transcript levels of ZO-1 (Fig. 4A). The immunoreactivity of ZO-1 (green) detected on cell surface after stimulation with 27OHChol was reduced by BFA (Supplementary data 3B). These results indicated that ZO-1 protein is transported onto cell surface of monocytes via by the ER-to-Golgi transport system.

### Involvement of multiple protein kinases in ZO-1 expression

**Figure 5:**
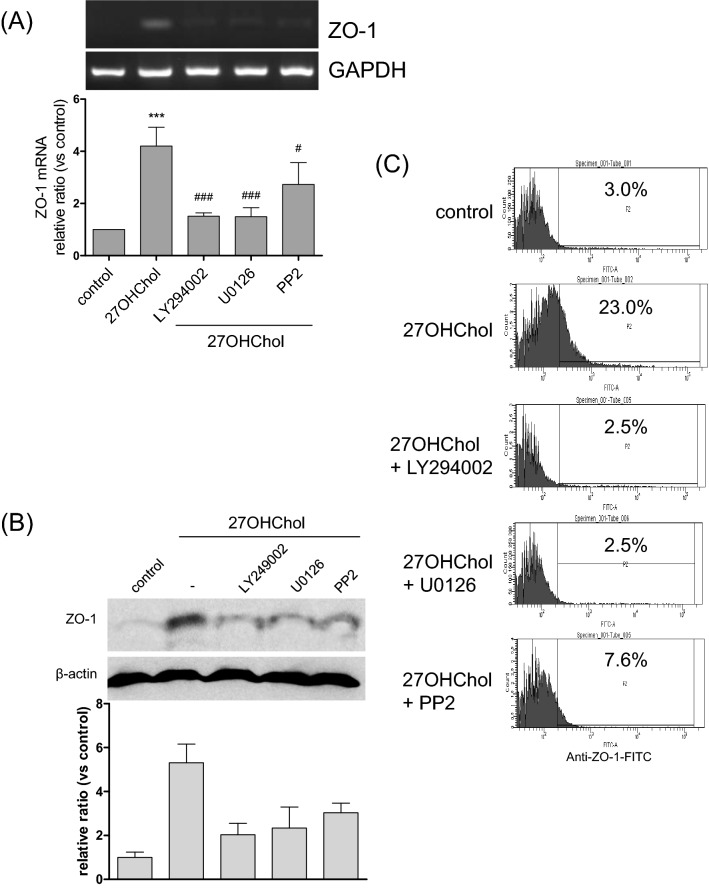
Effects of kinase inhibitors on the 27OHChol-induced ZO-1 expression. THP-1 cells (1 × 10^6^ cells/60 mm culture dish) were treated for 2 h with the indicated inhibitors (10 μM each) and stimulated for 48 h with 2.5 µg/ml of 27OHChol. (**A**) The levels of ZO-1 transcripts were analyzed using RT-PCR and real-time PCR. The data are expressed as the mean ± SD (n = 3 replicates/group). ***P < 0.01 versus control; ^###^*P* < 0.01 versus 27OHChol; ^#^*P* < 0.1 versus 27OHChol. (**B**) Total proteins obtained from the cells were analyzed via Western blot to detect ZO-1. (**C**) The ZO-1 protein on the cell surfaces was analyzed by flow cytometry. The results are representative of three independent experiments.

Multiple signaling pathways have been demonstrated to play roles in the cellular effects of 27OHChol. In this study, we investigated the involvement of the PI3K, ERK, and Src pathways in ZO-1 expression using specific inhibitors. An increase in the ZO-1 transcript levels was blocked in the presence of LY294002 (an inhibitor of PI3K) and U0126 (an inhibitor of MEK/ERK). PP2 (an inhibitor of the Src kinase family) also impaired the transcription of ZO-1 (Fig. 5A). In agreement with the RT-PCR and real-time PCR data, the levels of ZO-1 protein in the cells and on the cell surface were lowered in the presence of kinase inhibitors (Fig. 5B, C and Supplementary data 3C). We suggest that the inhibitory effects of the kinase inhibitors were not due to cytotoxicity because none of them decreased the cell viability (Supplementary data 2A). These results imply that the PI3K, ERK, and Src kinase pathways are necessary for 27OHChol to induce expression of ZO-1.

### Effects of drugs suppressing DC differentiation on ZO-1 expression

**Figure 6:**
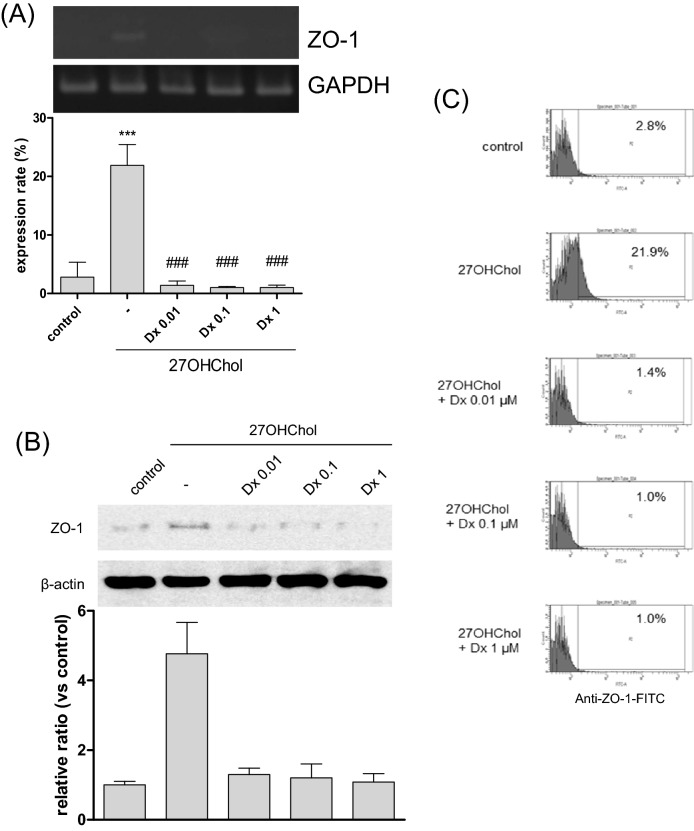
Effects of Dx on the 27OHChol-induced ZO-1 expression. THP-1 cells were stimulated with 2.5 µg/ml of 27OHChol in the presence of 0.01, 0.1, and 1 μM dexamethasone (Dx) for 48 h. (**A**) The levels of ZO-1 transcripts were analyzed by RT-PCR and quantitative real-time PCR. ***P < 0.01 versus control; ^###^*P* < 0.01 versus 27OHChol; ^#^*P* < 0.1 versus 27OHChol. (**B**) Proteins harvested from the cells were analyzed via Western blot to detect ZO-1. (**C**) The cells, treated with 27OHChol and indicated concentration of Dx, were immunostained with fluorescence-conjugated antibodies against ZO-1 and analyzed by flow cytometry. The results are representative of three independent experiments.

DC differentiation was suppressed by drugs such as cyclosporine A (CsA), diclofenac (Df), and dexamethasone (Dx)^8,12,13^. Therefore, we investigated the correlation between ZO-1 and DC differentiation by assessing the effects of these drugs on ZO-1 expression. The levels of ZO-1 transcripts, which were elevated following stimulation with 27OHChol, decreased in the presence of Dx (Fig. 6A). In agreement with the results of the RT-PCR and real-time PCR, Dx also suppressed the expression of ZO-1 protein (Fig. 6B). The expressed ZO-1 protein was confirmed using flow cytometry (Fig. 6C). The percentage of ZO-1 expression on the cell surface increased from 2.8 to 21.9% following treatment with 27OHChol, and this was reduced to 1.4%, 1.0%, and 1.0% by 0.01, 0.1, 1 μM of Dx, respectively. When visualized, ZO-1 protein (green fluorescence) was localized on the surface after stimulation with 27OHChol, but the fluorescence was barely detected in the presence of Dx (Supplementary data 3D). CsA and Df also affected the 27OHCHol-induced expression of ZO-1 at transcript and protein levels (Supplementary data 4). The three drugs did not cause cytotoxicity at the concentrations that impaired expression of ZO-1 (Supplementary data 2B). These results suggest that expression of ZO-1 coincides with DC differentiation in a milieu in which 27OHChol is abundant.

## Discussion

In this study, we demonstrated the increased expression of ZO-1 alone in monocytic cells following exposure to 27OHChol, indicating an induction of ZO-1 in response to an extracellular stimulus of oxidized cholesterol. ZO proteins, comprising ZO-1, ZO-2, and ZO-3, are ubiquitous scaffolding proteins. They are co-localized at junctional sites and can bind with themselves via the PDZ2 domain^14^. They display overlapping, but distinctive, expression patterns. ZO-1 is expressed at cell junctions of cardiac myocytes, but ZO-2 is not expressed in the heart^15^. ZO-2 and ZO-3 are concentrated in epithelial and endothelial tight junctions^14,16^. Although they share functional and structural similarities, ZO proteins display differential regulation and functions under hypercholesterolemic circumstances.

Besides the specific association of ZO-1 with tight junctions, it is involved in the regulation of the cell cycle. This protein has been detected in the nucleus during the proliferation of epithelial cells^17,18^. ZO-1 binds with ZO-1-associated nucleic acid-binding protein (ZONAB), promoting cell proliferation. ZO-1 functions as a suppressor of ZONAB and controls the accumulation of ZONAB in the nucleus by cytoplasmic sequestration; the overexpression of ZO-1 results in reduced nuclear ZONAB accumulation and proliferation^16,18^. We demonstrated that ZO-1 induced in the presence of 27OHChol is mainly localized on the cell surface. Collectively, these findings suggest that ZO-1 may regulate proliferation by sequestering the proteins involved in proliferation in the cytoplasm, in the presence of 27OHChol.

ZO proteins are multi-domain scaffolds that bind directly to various types of proteins, in addition to having scaffolding functions in organizing gap junction complexes, via the PDZ (PSD-95/Discs-large/ZO-1) domain, SH3 domain, and guanylate kinase domain^14^. ZO-1 can bind with protein kinases in the cytoplasm. After binding with the serine/threonine-protein kinase MRCKβ, the ZO-1/MRCKβ complex regulates migration at the leading edge of migrating cells^19^. Monocytic cells exposed to 27OHChol differentiate into DCs, which can migrate to inflammatory regions where they accelerate immune responses^7^. We demonstrated that CsA, Df, and Dx impair the ZO-1 expression induced by 27OHChol, and these drugs are well known for their anti-inflammatory and immunosuppressive effects^8,12,13^. Furthermore, the PI3K, ERK, and src kinases play crucial roles in migration^20–22^. Therefore, the results of this study suggest the possibility that the upregulated ZO-1 protein may be involved in cell migration, via interactions with protein kinases during inflammation or immune responses. Additionally, a study showed that the ZO-1 expression was affected through redox signaling pathway^23^. And, a recent study showed that oxysterol induced biological effects via redox signaling pathway^24^. These studies suggest a relationship of the ZO-1 expression induced by 27OHChol and NOX signals in the monocytic cells. However, the relationship does not well studied yet, and we need further studies about the signaling involvement.

Oxysterols exist as lipid-mixture in blood and tissues. A study reviewed that the mixed oxysterols played as immune-modulator in intestinal immunity^25^. To know synergic effects of the oxysterol-mixture, we examined with normal cholesterol and various oxysterols oxygenated by enzymatically (24sOHChol and 27OHChol) and non-enzymatically (7αOHChol, 7βOHChol and 7-ketocholesterol (7 K)). The ZO-1 expression of monocytic cells was only induced by the treatment of 27OHChol and 7αOHChol, and the other oxysterols were not affected. The ZO-1 expression by 7αOHChol was reported^11^. Co-treatment of the other oxysterols with 27OHChol was not showed negatively or positively synergic effects. Co-treatment of 7 K and 27OHChol was showed a toxic effect. These studies indicate that not all oxysterols influence to the TJP expression on monocytic cells.

In summary, we demonstrated that 27OHChol upregulates ZO-1 using ER-to-Golgi body transport system via PI3K, ERK, and src in monocytic cells, which is impaired by the drugs suppressing DC differentiation. These results also cause us to question the biological functions of the upregulated protein. Further investigations are needed to understand whether ZO-1 plays roles in migration and the regulation of cell proliferation during the process of DC differentiation.

## Materials and methods

### Cell culture and reagents

THP-1 cells purchased from the American Type Culture Collection (cat. no. TIB-20; ATCC, Manassas, VA, USA) were maintained in RPMI 1640 containing 10% fetal bovine serum (FBS) in the presence of penicillin and streptomycin. Brefeldin A (BFA) was purchased from Sigma-Aldrich (St. Louis, MO, USA). Cell Surface Protein Isolation Kit was purchased from Thermo Scientific (IL61101, USA). Membrane protein was harvested using Cholesterol and 27OHChol were purchased from Sigma-Aldrich and Santa Cruz Biotechnology (Santa Cruz, CA, USA), respectively, and dissolved in ethanol. Antibodies against ZO-1 (sc-10804; 220 kDa) and β-actin (sc-47778; 45 kDa) were purchased from Santa Cruz Biotechnology. Alexa Fluor 488-conjugated secondary antibody (A11059) for FACS analysis and immunofluorescence were purchased from Invitrogen (Eugene, Oregon, USA). Reagents for reverse transcription were purchased from Promega Corporation (Madison, WI, USA).

### Treatment of reagents

Concentrations of each reagent were followed; 2.5 μg/ml of 27OHChol, 10 μg/ml of BFA, 10 μM of kinase inhibitors, and 1 μM of Dx. And the treatments were incubated for 48 h.

### Reverse transcriptase and real-time polymerase chain reaction (RT and real-time PCR)

Total RNA was reverse transcribed at 42 °C for 1 h using 100 U Moloney Murine Leukemia Virus reverse transcriptase in a 10-μL reaction volume containing 50 mM Tris–HCl (pH 8.3 at 25 °C), 55 mM KCl, 3 mM MgCl_2_, 10 mM DTT, 1 μg oligo dT 15 primers, 0.125 mM each dNTP, and 40 U RNase inhibitor. Subsequent qPCR was performed following a previously described protocol^13^. Each 20-μL reaction mixture consisted of 10-μL SYBR Green Master Mix, 2-μL gene-specific forward and reverse primers (10 pM each), and a cDNA template. The thermal cycling conditions were: 95 °C for 10 min, followed by 45 cycles of 95 °C for 10 s, 50 °C for 10 s, and 72 °C for 10 s. Primer sequences for the real-time PCR were as follows. ZO-1: 5′- ccagcatcatcaacctctgc (forward) and 5′- catgcgacgacaatgatggt (reverse), ZO-2: 5′- agccccgagaacttttcttc (forward) and 5′- cttcttgaatgccagcaaca (reverse), ZO-3: 5′- catccaggagggagatcaga (forward) and 5′- ccagaaaatgtcctgcttcc (reverse), and GAPDH: 5′- atggggaaggtgaaggtcg (forward) and 5′- ggggtcattgatggcaacaata (reverse).

### Western blot analysis

Cells were lysed with lysis buffer (INTRON Biotechnology, Daejeon, Korea) containing a protease inhibitor cocktail (Sigma-Aldrich). Proteins in the lysates were separated by SDS-PAGE and transferred onto PVDF membranes. The membrane was separated for binding of ZO-1 (about 220 kDa) and β-actin (about 45 kDa) antibodies. After incubating for 1 h with 5% skim milk in 0.1% Tween 20/TBS to block the non-specific binding of primary antibodies, the separated membranes placed in each tray were probed with specific primary antibodies at 4 °C overnight. After three washes with the wash buffer (0.1% Tween 20/TBS) for 15 min each, membranes were incubated with HRP-conjugated secondary antibodies for 1 h at room temperature. Membranes were then washed a further three times using the wash buffer for 15 min each, and bands were detected using the enhanced chemiluminescence (ECL) Western blotting detection system (Thermo Scientific, IL, USA). Chemiluminescence images were captured using an Amersham Imager 600 (GE Healthcare Life Sciences, Pittsburgh, PA, USA).

### Immunofluorescence

THP-1 cells were seeded on cover slips (coated with 0.2% gelatin/PBS for 1 h) and stimulated for 48 h with cholesterol or 27OHChol. The cells were fixed for 20 min with 1% paraformaldehyde and incubated for 1 h with a blocking solution of 5% skim milk in PBS. After incubating for 2 h with the primary antibody against ZO-1 diluted in the blocking solution (1:100) at room temperature, the cells were washed twice for 5 min each using PBS. Following incubation for 1 h with secondary antibodies diluted in PBS (1:200) at room temperature in the dark, the cells were washed using PBS. The cover slips were mounted, and the cells were visualized using a confocal microscope (FV1000; Olympus Cor., Tokyo, Japan).

### Flow cytometric analysis

After harvesting by centrifugation at 200×*g* for 5 min at room temperature, both treated and untreated THP-1 cells were incubated for 2 h with anti-ZO-1 antibodies diluted to 1:100 in FACS buffer (2 mM EDTA and 0.2% BSA in PBS). The cells were washed with cold PBS and incubated for 1 h with fluorescent dye-conjugated secondary antibodies diluted to 1:200 in FACS buffer at 4 °C. After washing with cold PBS, the cells were resuspended in 1% paraformaldehyde in PBS. Subsequently, the cells were analyzed using a flow cytometer (FACS CANTO II; BD Company, NJ, USA).

### Statistical analysis

Statistical analyses were performed using one-way ANOVA, followed by Tukey’s multiple comparison tests, using GraphPad Prism (version 5.0; GraphPad software Inc., CA, USA).

## Supplementary Information

Supplementary Information.

## Data Availability

The datasets used and/or analysed during the current study available from the corresponding author on reasonable request.
