# Supplementary material for: 27-Hydroxycholesterol induces expression of zonula occludens-1 in monocytic cells via multiple kinases pathways
Source: Sci Rep. 2022 May 17;12:8213. doi: 10.1038/s41598-022-12416-w (PMC9114403; doi:10.1038/s41598-022-12416-w)
Supplement: Supplementary file 1 — Supplementary Information. [file 41598_2022_12416_MOESM1_ESM.docx]

**Supplementary data 1**

Role of 27OHChol in expression of ZO-family on monocytic cells

THP-1 cells (1×10^6^ cells/60 mm culture dish) were stimulated for 48 h with 5 µg/ml of cholesterol or 2.5 µg/ml of 27OHChol. Total RNAs were extracted and analyzed by quantitative real-time PCR. Results are representative of three independent experiments. ***: *P* < 0.01 vs. control

**Supplementary data 2**

(A)

(B)

Check of viability for toxicity of inhibitors and drugs on monocytic cells

THP-1 cells (1×10^4^ cells/96 well plate; triplicate) were co-cultured for 48 h with 2.5 µg/ml of 27OHChol and the experimented concentration of inhibitors/drugs. Viability of the cells was determined with cell counting kit-8 (CCK-8).

**Supplementary data 3**

**
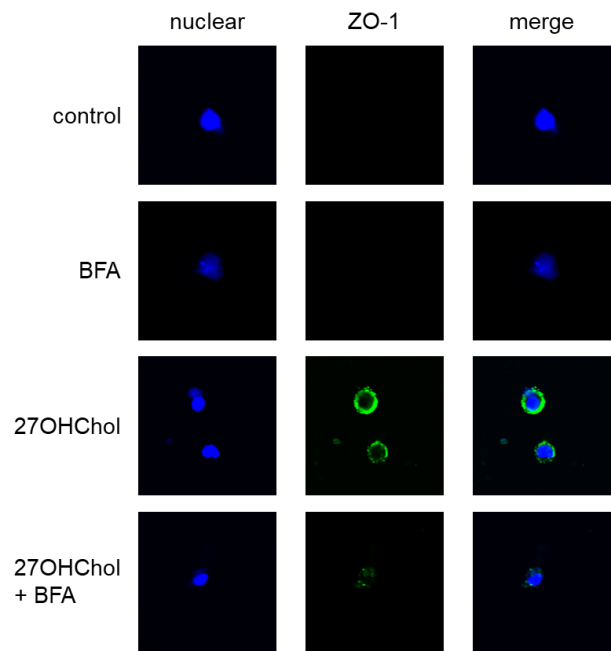

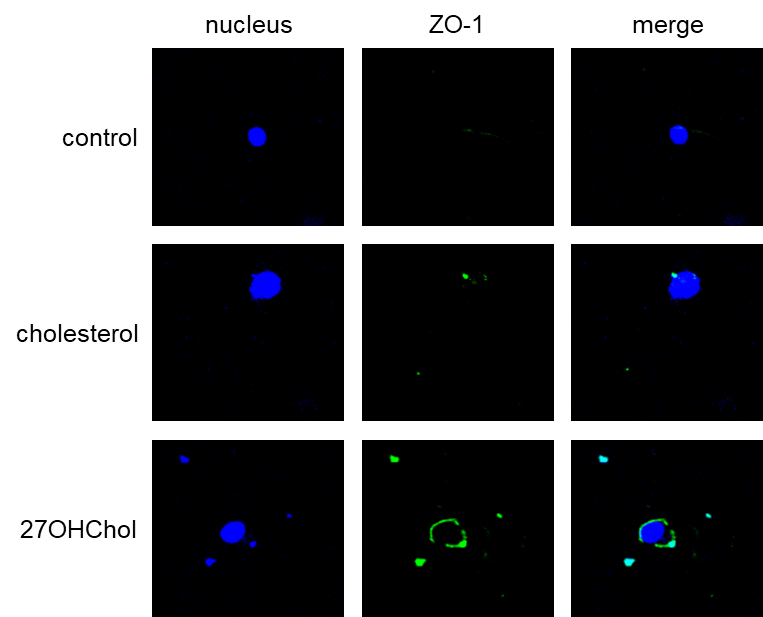
**

(B)

(A)

**
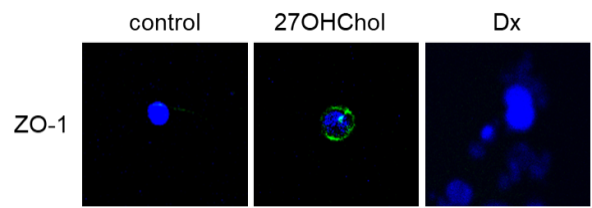

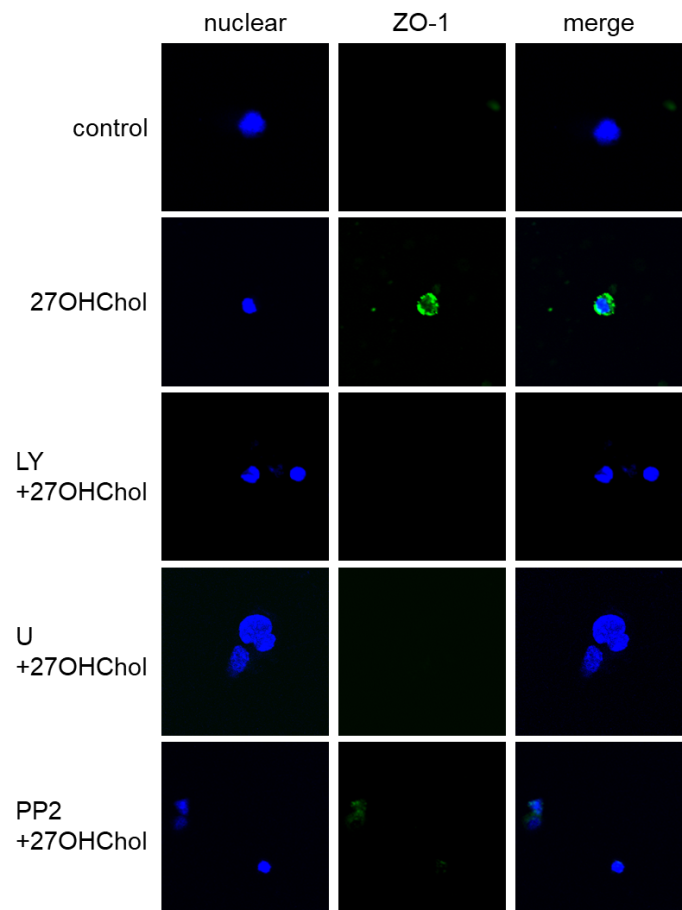
**

(D)

(C)

Results of immunofluorescence of the ZO-1 expression in the 27OHChol-stimulated monocytic cells

THP-1 cells (1×10^6^ cells cultured on coverslip) were treated with 2.5 µg/ml of 27OHChol and indicated condition 48 h. ZO-1 was labeled with a green-fluorescent dye, and the nuclei were counterstained with DAPI. And the fluorescence was visualized via confocal microscopy. (A) The cells were treated with cholesterol and 27OHChol. (B) The cells were treated with 10 µg/ml of BFA and 27OHChol. (C) The cells were pre-treated with 10 µM of indicated inhibitors for 2 h, and were treated with 27OHChol. (D) The cells were treated with 1 µM of Dx and 27OHChol. The results are representative of three independent experiments.


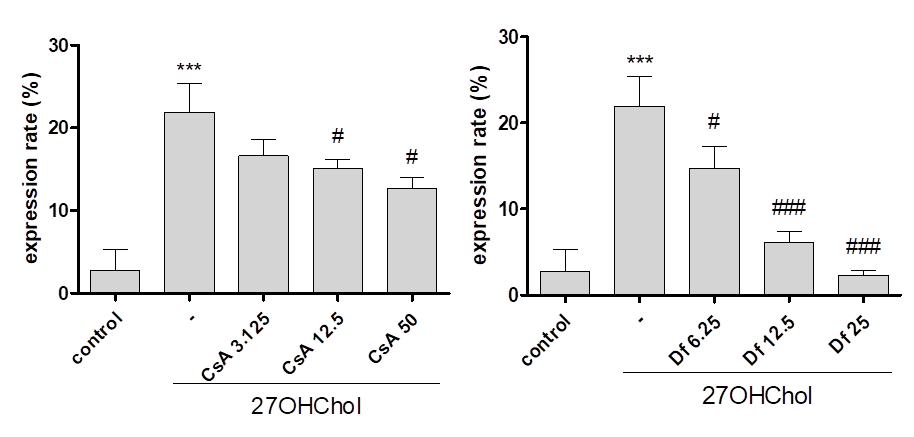
**Supplementary data 4**

(A)

(B)


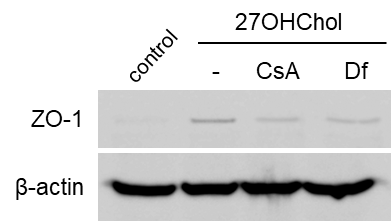


(C)


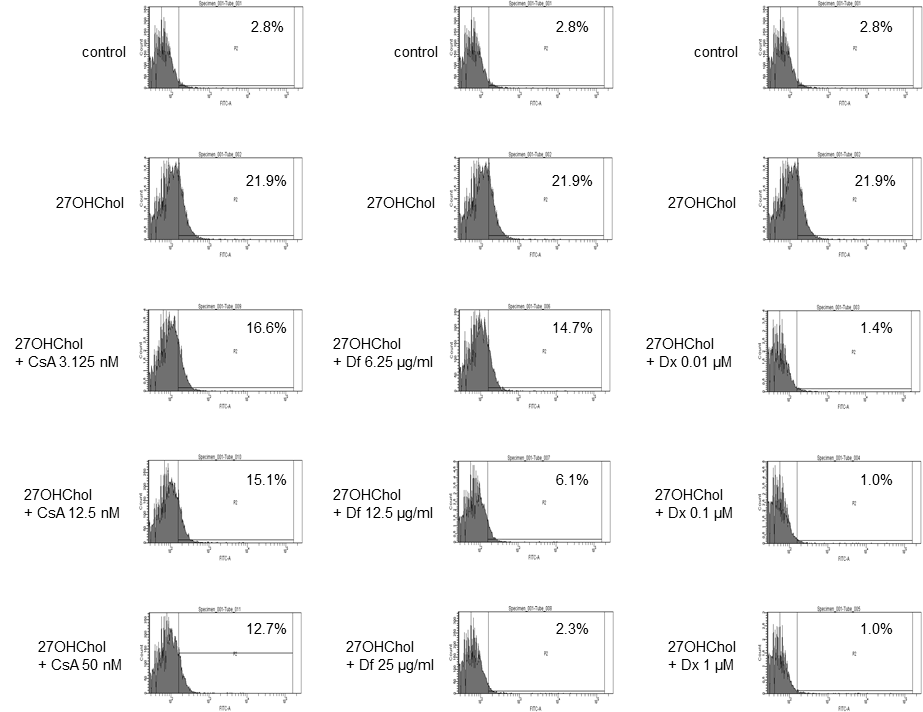


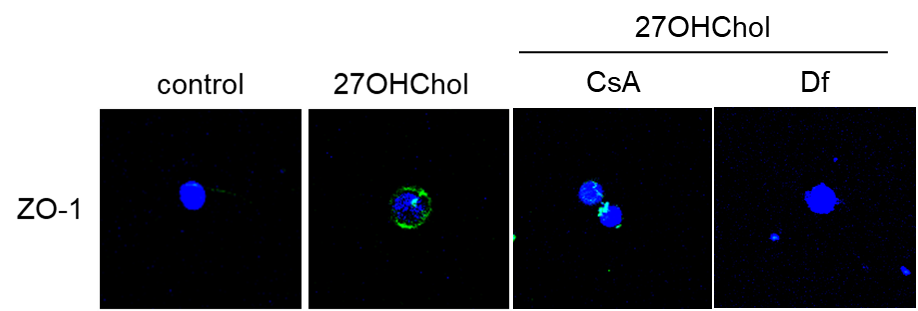
(D)

Role of differentiation-involved drugs in the 27OHChol-induced ZO-1 expression on monocytic cells

THP-1 cells (1×10^6^ cells/60 mm culture dish) were treated with 2.5 µg/ml of 27OHChol and 50 nM of CsA/ 25 μg/ml of Df for 48 h. (A) Transcription of ZO-1 was analyzed by real-time PCR. ***: *P* < 0.01 vs. control; #: *P* < 0.1 vs. 27OHChol; ###: *P* < 0.01 vs. 27OHChol (B) Protein level of the ZO-1 was determined by Western blot. (C) The cells, stimulated with 27OHChol and indicated concentration of drugs, were immunostained with antibody against ZO-1 and were analyzed by flow cytometry. (D) The cells cultured on coated coverslip were immunostained with anti-ZO-1 antibody, and were visualized by confocal microscopy. Results are representative of three independent experiments.
